# Intergenerational transmission of anxiety: A comparison of transdiagnostic and disorder‐specific pathways

**DOI:** 10.1002/jcv2.70117

**Published:** 2026-05-25

**Authors:** Hyunsik Kim, Lifang Pan, Ardesheer Talati, Marc J. Gameroff, Myrna M. Weissman, Helen Blair Simpson

**Affiliations:** ^1^ Department of Psychology Sogang University Seoul South Korea; ^2^ New York State Psychiatric Institute New York New York USA; ^3^ Department of Psychiatry Columbia University Irving Medical Center and Vagelos College of Physicians and Surgeons Columbia University New York New York USA; ^4^ Division of Translational Epidemiology & Implementation Science New York State Psychiatric Institute New York New York USA; ^5^ Department of Epidemiology Columbia University Mailman School of Public Health New York New York USA; ^6^ Department of Psychiatry Columbia University New York New York USA

**Keywords:** anxiety disorders, childhood anxiety onset, Hierarchical Taxonomy of Psychopathology, intergenerational transmission, internalizing psychopathology, parental psychopathology, structural equation modeling, survival analysis, transdiagnostic factors

## Abstract

**Background:**

Individuals with a parental history of anxiety disorders are at risk of developing an anxiety disorder. Prior studies have focused on the transmission of specific anxiety disorders across generations (i.e., disorder‐specific transmission), often overlooking the possibility that a general tendency to develop various forms of anxiety might be passed from parents to offspring (i.e., transdiagnostic transmission). Our study aimed to investigate whether (a) anxiety disorders are transmitted intergenerationally through disorder‐specific or transdiagnostic pathways and (b) different parental transdiagnostic factors differentially predict the offspring's anxiety and the onset of childhood anxiety disorders.

**Methods:**

Data were drawn from a subset of participants within a multigenerational family study, focusing specifically on 211 parents (generation 2; G2) and 253 children (generation 3; G3) from families where the first‐generation probands either had a diagnosis of moderate/severe major depressive disorder or no lifetime psychiatric disorders. Structural equation modeling was employed to test transdiagnostic and disorder‐specific pathways of anxiety transmission, and to compare different G2 transdiagnostic factors in predicting the G3 anxiety factor. Survival analysis was used to examine how different G2 factors predicted the onset of childhood anxiety disorders in G3.

**Results:**

The results indicated that parents transmit general vulnerabilities for developing various forms of anxiety disorders to their offspring (*β*s = 0.337–0.412, *ps* = 0.012–0.040), rather than vulnerabilities for specific types of anxiety disorders. Different parental transdiagnostic factors (internalizing, anxiety, and distress) significantly and similarly predicted both the offspring anxiety factor (*β*s = 0.400–0.469, *ps* = 0.006 to < 0.001) and the onset age of childhood anxiety disorders (*HR*s = 2.11–2.71, *ps* < 0.05).

**Conclusion:**

These results underscore the importance of considering transdiagnostic factors in understanding the intergenerational transmission of anxiety. Targeting these broad underlying vulnerabilities may enhance the effectiveness of interventions and prevention strategies. Future research should further explore potential transdiagnostic genetic, neurobiological, and environmental mechanisms involved in intergenerational anxiety transmission.

## Key Points

**What's known?**

- Anxiety disorders run in families, but prior studies have mainly focused on disorder‐specific transmission.
- Transdiagnostic models suggest that broad underlying vulnerabilities—rather than discrete diagnoses—may better account for intergenerational risk.

**What's new?**

- This study is the first to compare transdiagnostic versus disorder‐specific pathways of anxiety transmission across generations.
- Anxiety was transmitted intergenerationally primarily through a transdiagnostic pathway, rather than through disorder‐specific mechanisms.
- Parental transdiagnostic factors (internalizing, anxiety, distress) significantly and similarly predicted both children's anxiety factor and the onset of childhood anxiety disorders.

**What's relevant?**

- These findings support the utility of transdiagnostic frameworks in understanding how anxiety is transmitted intergenerationally.
- Targeting broad underlying vulnerabilities in parents may improve early identification and prevention efforts for childhood anxiety.

## INTRODUCTION

Anxiety disorders as a group (e.g., social anxiety disorder, panic disorder) are among the most common psychiatric conditions, with 28.8% of American adults experiencing an anxiety disorder in their lifetime (Kessler et al., 2005). Individuals with anxiety disorders often face significant adverse life outcomes, including impaired job performance (Wald, 2010), academic impairments (Nail et al., 2015), lower quality of life (Olatunji et al., 2007), and poor social functioning (Olatunji et al., 2007; Settipani & Kendall, 2013). The high prevalence and treatment costs of these conditions impose an enormous financial burden on society (Leon et al., 1995; Marciniak et al., 2005). Thus, understanding the causes of these disorders is crucial, as it can inform early intervention and prevention strategies that have the potential to reduce the individual and societal burden of anxiety.

Anxiety runs in families (Rapee, 2012), with individuals having a parental history of anxiety disorders more likely to suffer from these disorders themselves (Barlow, 1988; Fyer et al., 1990; Harris et al., 1983; Schreier et al., 2008; Turner et al., 1987). Indeed, prior research has indicated significant familial aggregation for *various* anxiety disorders, including simple phobia, social phobia, agoraphobia, panic disorder, generalized anxiety disorder (GAD), and obsessive‐compulsive disorder (OCD) (Fyer et al., 1990, 1995; Hettema et al., 2001; Noyes Jr et al., 1987). Additionally, some studies have suggested distinct transmission patterns for specific anxiety disorders, such as panic disorder compared to GAD (Noyes Jr et al., 1987).

At the same time, given the high comorbidity and partial symptom overlap between different forms of anxiety disorders (Kessler et al., 2005; Kim & Eaton, 2015; Kotov et al., 2017), it is possible that anxiety transmission involves a *general propensity* for developing various forms of anxiety, rather than propensity for developing a *particular* form. Indeed, several studies have suggested the potential transmission of a general propensity for anxiety (Andrews et al., 1990; Barlow, 1988; Rosenbaum et al., 1991; Schreier et al., 2008). These studies collectively imply that a broad, heritable tendency toward anxiety, which may manifest in various anxiety disorders depending on individual and environmental contexts, could be transmitted across generations. For example, in a longitudinal survey of a community sample in Germany (comprising 933 mother‐child pairs), Schleier et al. (2008) conducted a survival analysis to examine the likelihood of offspring developing any anxiety disorder based on the mother's specific anxiety disorder diagnosis. Results showed that having a mother with an anxiety disorder, compared to having a mother without one, was associated with an increased risk of developing *any* anxiety disorder.

This aligns with growing evidence for a transdiagnostic conceptualization of psychopathology (Kotov et al., 2017; Krueger & Eaton, 2015; Krueger et al., 2018). This approach posits that several transdiagnostic dimensions cut across diagnostic boundaries, accounting for the commonality and comorbidity among different psychiatric disorders. Prior literature on these transdiagnostic factors has demonstrated that (a) they largely account for the onset and homotypic continuity of psychiatric disorders (Kessler et al., 2011) and predict subsequent mental disorder outcomes (Kim & Eaton, 2015), (b) these transdiagnostic factors contribute to various forms of child and adolescent psychopathology and are highly heritable (Tackett et al., 2013), and (c) internalizing disorders in parents are strongly associated with similar patterns of psychopathology in their offspring, suggesting transdiagnostic family factors that may contribute to internalizing problems in youth (Schleider & Weisz, 2017).

Taken together, evidence for both familial specificity and transdiagnostic liability has shaped two complementary lines of research on the intergenerational transmission of anxiety disorders. Some studies have focused on the familial aggregation of specific anxiety disorders, providing evidence for disorder‐specific parent–offspring associations (Fyer et al., 1990, 1995; Low et al., 2008; Steinhausen et al., 2016). In contrast, a growing body of work has adopted transdiagnostic approaches, showing that familial risk often extends across diagnostic boundaries and may be partly attributable to shared transdiagnostic liability, as demonstrated in cohort, registry, and meta‐analytic studies (Chen et al., 2025; Starr et al., 2014; Uher et al., 2023; Zhou et al., 2023).

However, relatively few studies have directly compared transdiagnostic and disorder‐specific pathways of transmission within the same analytic framework. Notably, Starr et al. (2014) found that offspring internalizing outcomes were primarily predicted by a broad transdiagnostic internalizing factor rather than by specific parental diagnoses; however, this work focused on a higher‐order internalizing construct rather than anxiety‐specific latent factors and did not evaluate the relative contributions of multiple transdiagnostic dimensions. Moreover, a recent large‐scale meta‐analytic study (Uher et al., 2023) demonstrated that intergenerational transmission of mental disorders is both transdiagnostic and partially disorder‐specific at the level of observed diagnoses. Although informative, this work relied on aggregated diagnostic outcomes and therefore could not determine whether disorder‐specific transmission persists after accounting for shared transdiagnostic liability. By using latent variable modeling within a multigenerational high‐risk cohort, the present study directly disentangles transdiagnostic and anxiety‐specific pathways, thereby clarifying the mechanisms underlying intergenerational anxiety transmission.

Previous research has identified several transdiagnostic factors that contribute to a broad susceptibility to anxiety and mood disorders: (a) anxiety (general tendencies toward anxiety disorders), (b) distress (general tendencies toward mood disorders), and (c) internalizing (broad vulnerability to internalizing disorders like anxiety and depression). Studies have shown that a transdiagnostic internalizing factor explains correlations among internalizing disorders (Kotov et al., 2017; Krueger & Eaton, 2015; Krueger et al., 1998, 2018; Krueger & Markon, 2006), particularly between anxiety and mood disorders (Clark & Watson, 1991; Eaton et al., 2013). This internalizing factor further bifurcates into anxiety (also known as fear) and distress factors (Kim & Eaton, 2015; Krueger & Markon, 2006). Given this hierarchical structure and the high correlation between anxiety and mood disorders, exploring how these transdiagnostic factors predict anxiety transmission could provide valuable insights into their comparative predictive validity.

To address this research gap, we utilized data from a multigenerational study that followed the children and grandchildren of probands with either primary moderate/severe major depressive disorder (MDD) or no lifetime psychiatric disorders. Although the study primarily focused on MDD, it is relevant to our research on the intergenerational transmission of anxiety due to (a) the study's unique transgenerational design, (b) the inclusion of comprehensive assessments of anxiety and mood disorders (assessed using by the Schedule for Affective Disorders and Schizophrenia), and (c) the high comorbidity between MDD and anxiety disorders, which resulted in a significant proportion of the second and third generations being diagnosed with anxiety disorders. Indeed, this dataset has also been utilized in studies that investigated the development and heritability of anxiety disorders (Bushnell et al., 2020; Guffanti et al., 2016).

Our primary aim was to investigate whether anxiety disorders are transmitted via a disorder‐specific or transdiagnostic pathway. Based on the literature, we hypothesized that the parental (i.e., second‐generation) transdiagnostic anxiety factor, rather than disorder‐specific anxiety factors, would predict the offspring (i.e., third‐generation) anxiety factor. Given the high correlation and significant genetic overlap between various anxiety and mood disorders (Clark & Watson, 1991; Eaton et al., 2013; Tao et al., 2024), our secondary aim was to examine whether different parental transdiagnostic factors—anxiety, distress, and internalizing—differentially predict the offspring's anxiety factor. Finally, we used survival analysis to explore whether children of parents with elevated levels of these transdiagnostic factors are at increased risk for developing any anxiety disorder during childhood.

## METHODS

### Participants

We used data from a multigenerational study (1982–2020) that followed the children and grandchildren of adults (probands; first generation; G1) who either (a) had a primary diagnosis of moderate to severe MDD or (b) were from the same community but had no MDD or any other lifetime psychiatric disorder (Weissman et al., 1997, 2006, 2016). Offspring and later grandchildren were assessed at years 0, 2, 10, 20, 25, and 30. Offspring and grandchildren of probands with MDD constituted the MDD high‐risk group; offspring and grandchildren of probands without MDD constituted the low‐risk group. This study was reviewed and approved by the Institutional Review Board of the New York State Psychiatric Institute/Columbia University.

The current study focused on the biological offspring (i.e., second generation; G2; parents) and grandchildren (i.e., third generation; G3; children) of the probands. Data collection began at year 0 for G2 and at year 10 for G3. We included G2 parents and their G3 offspring for whom G3 had at least some childhood (ages 0–17 years) anxiety disorder diagnostic information available, along with corresponding demographic and clinical data for G2 parents. Of the 253 G3 participants in the analytic sample, 165 (65.22%) had complete childhood anxiety diagnostic histories through age 17, whereas 88 (34.78%) had incomplete histories due to discontinuation before age 18^1^; these individuals were treated as right‐censored at their last observed age in Cox regression models. Data from G2 (*n* = 211, mother = 120, father = 91), including both the biological offspring of G1 (*n* = 128) and married‐in's (*n* = 83) and G3 (*n* = 253, female = 132) were included in the analyses (Table 1). All 253 G3 participants had clinical data available for at least one G2 parent who was a biological descendant from the original G1 proband.

**TABLE 1:** Demographic and clinical characteristics of the sample.

Variables	Second generation (G2; parents)Total *n* = 211	Third generation (G3; children)Total *n* = 253
Female	120 (56.87)	132 (52.17)
Psyciatric disorders		
MDE	115 (54.50)	34 (13.44)
Dysthymia	75 (35.55)	18 (7.11)
Bipolar disorder	34 (16.11)	5 (1.98)
GAD	37 (17.54)	9 (3.56)
Specific phobia	52 (24.64)	56 (22.13)
Social phobia	29 (13.74)	26 (10.28)
Panic disorder	35 (16.59)	13 (5.14)
Separation anxiety	31 (14.69)	16 (6.32)
OCD	10 (4.74)	7 (2.77)
Agoraphobia	10 (4.74)	3 (1.19)
PTSD	16 (7.58)	12 (4.74)
Any anxiety disorder	95 (45.02)	95 (37.55)
Any mood disorder	115 (54.50)	58 (22.92)
Mean age at giving birth to an offspring, mother (SD)	28.53 (5.42)	N/A
Mean age at giving birth to an offspring, father (SD)	29.66 (5.75)	N/A
Mean age at first interview (SD)	N/A	10.94 (4.82)
Mean age at last interview (SD)	N/A	21.43 (9.30)

*Note*: G2 participants included both the biological offspring of G1 probands (*n* = 128) and their spouses who married into the families (*n* = 83).

Abbreviations: G2, generation 2, parents; G3, generation 3, children; GAD, generalized anxiety disorder; MDE, major depressive episode; OCD, obsessive‐compulsive disorder; PTSD, post‐traumatic stress disorder.

### Assessments

#### Anxiety disorders

Anxiety disorders were assessed at each assessment time point using the Schedule for Affective Disorders and Schizophrenia‐Lifetime version (SADS‐LA) for adults (Mannuzza et al., 1986) and the Kiddie‐SADS (K‐SADS) for children aged 6–17 years. Specifically, K‐SADS‐e (Orvaschel et al., 1982) was used in wave 3 and K‐SADS‐PL (Kaufman et al., 1997) was used in waves 4 through 6. In the diagnostic interviews, participants were asked to report on psychiatric symptoms experienced since the last interview, or since birth if there had been no previous interview. Interviewers were blinded to the previously‐collected clinical information of all participants.

Anxiety disorders included GAD, specific phobia, social phobia, panic disorder, separation anxiety disorder, OCD, agoraphobia, and posttraumatic stress disorder (PTSD). PTSD assessments were incorporated starting at the 20‐year follow‐up mark, which corresponds to Wave 4 of the study. We included anxiety disorder diagnoses assessed as definite or probable using a “best estimate” method; definite diagnoses were assigned when all diagnostic criteria for an anxiety disorder were met, and probable diagnoses were assigned when more than half of the major diagnostic criteria were met. This process was conducted by experienced mental health professionals who had access to all available information, were not involved in the interviews, and were blind to the clinical status of previous generations (Leckman et al., 1982).

#### Additional assessments of childhood internalizing disorders

We included assessments of other internalizing disorders with typical onsets relatively early in life, including major depressive episode (MDE), dysthymia, and bipolar disorder. Psychiatric conditions were classified as childhood onset if the first episode's onset was prior to age 18. These diagnoses used the best‐estimate procedure described above.

### Analysis

All analyses were conducted controlling for sex, age at first interview, high‐risk status (i.e., offspring of probands with MDD vs. offspring of adults with no MDD or psychiatric history), as well as for intraclass correlation (ICC). Since some G3 children shared the same G2 parent, the data had a nested structure. To account for the non‐independence of observations due to family clustering, we structured our analyses using the “Cluster” and “Type = Complex” options in Mplus, specifying family as the clustering variable (Muthén & Muthén, 2017). This approach adjusted standard errors and model fit statistics to account for ICC, ensuring more accurate statistical inference. The inclusion of covariates and adjustment for ICC were informed by: (a) degree of sibling similarity in psychiatric conditions within families (Kendler et al., 1992; van Sprang et al., 2023), (b) gender and age differences in developmental trajectories of anxiety disorder symptoms during adolescence (Hale III et al., 2008; Ohannessian et al., 2017), and (c) prior findings that children of parents with MDD had an increased likelihood of belonging to a persistent trajectory of childhood anxiety disorders (Bushnell et al., 2020).

Disorder diagnoses with frequency below 3% in either generation (bipolar disorder, OCD, and agoraphobia) were excluded to avoid model convergence issues. PTSD was excluded because it was not assessed until the 20‐year follow‐up and its inclusion significantly worsened the model fit. All analyses except the survival analysis were conducted using the weighted least squares mean and variance adjusted estimator, which is a robust estimator that accounts for non‐normality and is particularly well‐suited for modeling categorical data (Brown, 2015). For Cox regression, we used maximum likelihood estimation with robust standard errors (MLR), which implements full information maximum likelihood to handle missing data under standard assumptions in Mplus. SEM model fit was assessed using the comparative fit index (CFI; Bentler, 1990), the Tucker‐Lewis index (TLI; Tucker & Lewis, 1973), and the root mean square error of approximation (RMSEA; Steiger, 1990). CFI and TLI values ≥ 0.95 indicate good fit (Hu & Bentler, 1999), while values ≥ 0.90 are also considered acceptable (Bentler & Bonett, 1980; Marsh et al., 2004)). RMSEA values ≤ 0.06 indicate a good fit (Hu & Bentler, 1999).

### Testing the transdiagnostic and disorder‐specific pathways of anxiety transmission (primary aim)

To test the pathways, we modeled two types of factors and included them in SEM: (a) a transdiagnostic anxiety factor, represented by the shared variance among various anxiety disorders, and (b) a disorder‐specific factor, represented by the unique variance of a given anxiety disorder, thereby allowing direct comparison of transdiagnostic versus disorder‐specific pathway within the same analytic framework. The detailed steps of the modeling process are outlined below.

#### Modeling a transdiagnostic anxiety factor

To test the transdiagnostic pathway, we modeled a transdiagnostic anxiety factor for G2 and G3 by including binary diagnoses of specific phobia, social phobia, panic disorder, and separation anxiety disorder as indicators based on prior literature (Eaton et al., 2015). To ensure a valid comparison between G2 and G3 anxiety factors, we included the same set of anxiety disorder diagnoses for both generations. To facilitate interpretation, we standardized the transdiagnostic factor, setting its mean to 0 and variance to 1, so that SEM results reflect variations in standard deviation units rather than measurement units.

#### Modeling each anxiety disorder‐specific factor

To test each anxiety‐disorder specific pathway, we extracted the unique variance of each anxiety disorder (i.e., the residual variance after accounting for the shared variance among different anxiety disorders) and included it in the analysis. For example, the unique variance for panic disorder was characterized by the residual variance after accounting for the variance panic disorder shared with other disorders.

#### Testing the transdiagnostic and disorder‐specific pathways

**FIGURE 1:**
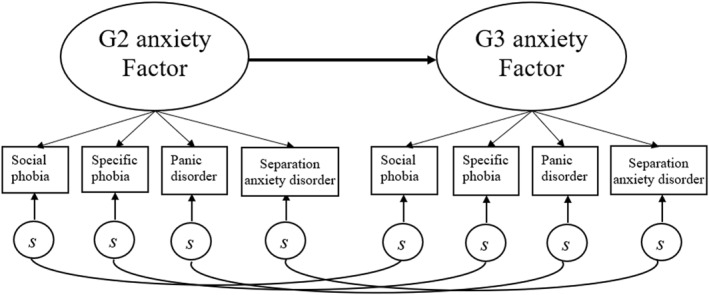
Graphic illustration of testing the transdiagnostic and disorder‐specific pathways of anxiety transmission. G2 = generation 2; s = disorder‐specific variance.

To test both pathways, we conducted a series of SEM analyses that (a) regressed the G3 anxiety factor on the G2 anxiety factor and (b) regressed a given anxiety disorder‐specific factor for G3 on the corresponding disorder‐specific factor for G2 in the same model. Accordingly, separate SEMs were estimated for each anxiety disorder (specific phobia, social phobia, panic disorder, and separation anxiety disorder), with each model simultaneously including both (a) the transdiagnostic pathway and (b) each disorder‐specific pathway; this approach reduced model complexity and avoided multicollinearity among residualized indicators, yielding a total of four analyses (Figure 1).

### Comparative analysis of different parental transdiagnostic factors in predicting the offspring anxiety factor (secondary aim)

After testing transdiagnostic and disorder‐specific pathways, we further examined how different parental transdiagnostic factors uniquely predicted the offspring anxiety factor. We modeled three transdiagnostic factors for G2: internalizing, distress, and anxiety, and an anxiety factor for G3. The G2 internalizing factor was modeled by including binary diagnoses of MDE, dysthymia, bipolar disorder, GAD, specific phobia, social phobia, panic disorder, and separation anxiety disorder, based on previous literature (Kotov et al., 2015). G2 distress factor was modeled by including binary diagnoses of MDE, dysthymia, bipolar disorder, and GAD based on prior literature (Eaton et al., 2015). The G3 anxiety factor included binary diagnoses of specific phobia, social phobia, panic disorder, and separation anxiety disorder, as described above. All transdiagnostic factors were standardized to facilitate interpretation.

We conducted a series of structural equation modeling (SEM) analyses in which the G3 anxiety factor was regressed on the G2 internalizing, anxiety, and distress factors one at a time. We further conducted a series of z‐tests, testing for significant differences in standardized path coefficients from different models, to examine the comparative validity of different parental transdiagnostic factors in predicting children's anxiety factor.

### Comparative analysis of different G2 transdiagnostic factors in predicting the onset of any childhood anxiety disorder in G3 (third aim)

#### Survival analysis

Survival analysis encompasses a set of statistical procedures used to estimate the survival function, representing the probability that an individual will survive (or not experience the outcome of interest) beyond a given temporal point (Klein et al., 2014). We used survival analysis to (a) estimate the probability of developing an anxiety disorder during childhood, and (b) examine how different parental transdiagnostic factors differentially predict the probability of developing any childhood anxiety disorder in offspring. Unlike SEM, which models associations between latent constructs, survival analysis provides hazard ratios (HR) that quantify how much the likelihood of anxiety disorder onset increases in response to an one‐unit increase in parental transdiagnostic factor levels.

We utilized the semi‐parametric Cox regression model, which makes fewer assumptions about survival function than other parametric models (e.g., Weibull, exponential) (Bradburn et al., 2003; Collett, 2023), making it a practical and appropriate choice. Conducting a series of Cox regression models, we regressed the age of onset of any anxiety disorder in G3 on the different transdiagnostic factors for G2, one at a time. We additionally conducted z‐tests to examine significant differences in HR obtained from different Cox regression models, thereby assessing the comparative predictive validity of various transdiagnostic factors. Transdiagnostic factors were standardized to facilitate interpretation. All analyses were performed using Mplus version 8.0.

## RESULTS

### Sample characteristics

Table 1 summarizes the sample characteristics. Of the G2 parents (*n* = 211), 120 (56.9%) were female. Among them, 128 (female = 59.4%) were biological offspring of generation 1 (G1), and 83 (female = 53.0%) were married in; 45.0% had ≥1 anxiety disorder and 54.5% had ≥1 mood disorder. The mean age for G2 parent parturition of a G3 offspring was 28.5 (SD = 5.4, range = 16.77–43.75) years for mothers and 29.6 (SD*=*5.8, range = 15.78–53.87) years for fathers.

Of the G3 offspring (*n* = 253), 132 (52.2%) were female. The mean age at first interview was 10.9 years (SD = 4.8, range = 3.8–35.40), and the mean age at last interview was 21.43 years (SD = 9.3, range = 4.4–45.14). 37.5% had at least one anxiety disorder, and 22.9% had at least one mood disorder.

### Testing the transdiagnostic and disorder‐specific pathways of anxiety transmission (primary aim)

The goodness‐of‐fit statistics for the SEM analyses examining the transdiagnostic and disorder‐specific pathways of anxiety transmission indicated acceptable to good model fit, with CFI and TLI values ranging from 0.854 to 0.952, and all RMSEA values below 0.05 (Table 2), which was consistent with expectations for models of this complexity and sample size (Wolf et al., 2013). Detailed results from these models are presented in Table 3. As Table 3 shows, the G2 anxiety factor significantly and consistently predicted the G3 anxiety factor across all four analyses (*β′s* = 0.337–0.412, SE*'s* = 0.161–0.165, *p's* = 0.012–0.04), whereas none of the G2 disorder‐specific factors significantly predicted the corresponding G3 disorder‐specific factors.

**TABLE 2:** Model fit index values of structural equation modeling and survival analysis for parental factors predicting offspring anxiety.

G2 predictors	G3 outcomes	RMSEA	CFI	TLI	AIC	BIC	SaBIC
Testing the transdiagnostic and disorder‐specific pathways of anxiety transmission using SEM							
Anxiety factor versus panic disorder‐specific component		0.015	0.946	0.928	N/A		
Anxiety factor versus separation anxiety disorder‐specific component		0.022	0.893	0.858			
Anxiety factor versus social phobia‐specific component		0.022	0.891	0.854			
Anxiety factor versus specific phobia‐specific component		0.014	0.952	0.936			
Comparing validity of different parental transdiagnostic factors in predicting offspring's anxiety factor using SEM							
Internalizing factor	Anxiety factor	0.023	0.855	0.828	N/A		
Anxiety factor		0.018	0.920	0.896			
Distress factor		0.017	0.898	0.867			
Comparing the validity of parental transdiagnostic factors in predicting the onset of childhood anxiety using survival analysis							
Internalizing factor	Childhood onset of any anxiety disorder	N/A			3554.393	3656.861	3564.926
Anxiety factor					1699.928	1742.329	1704.287
Distress factor					1761.387	1800.254	1765.382

Abbreviations: AIC, Akaike's information criterion; BIC, Bayesian information criterion; CFI, comparative fit index; G2, generation 2, parents; G3, generation 3, children; RMSEA, root mean square error of approximation; SaBIC, sample size‐adjusted Bayesian information criterion; SEM, Structural equation modeling; TLI, Tucker‐Lewis index.

**TABLE 3:** Results of the structural equation modeling analyses for parental factors predicting offspring anxiety.

G2 predictors	G3 outcomes	Beta	SE	*p*
Testing the transdiagnostic and disorder‐specific pathways of anxiety transmission				
G2 anxiety factor	G3 anxiety factor	0.337	0.164	0.04
G2 panic‐specific component	G3 panic‐specific component	0.676	0.672	0.314
G2 anxiety factor	G3 anxiety factor	0.409	0.163	0.012
G2 separation anxiety‐specific component	G3 separation anxiety‐specific component	−0.011	0.223	0.962
G2 anxiety factor	G3 anxiety factor	0.412	0.165	0.012
G2 social phobia‐specific component	G3 social phobia‐specific component	−0.044	0.177	0.802
G2 anxiety factor	G3 anxiety factor	0.371	0.161	0.021
G2 specific phobia‐specific component	G3 specific phobia‐specific component	0.241	0.136	0.077
Comparing validity of different parental transdiagnostic factors in predicting offspring's anxiety factor				
Internalizing factor	Anxiety factor	0.469	0.111	0.000
Anxiety factor		0.400	0.147	0.006
Distress factor		0.431	0.127	0.001

Abbreviations: G2, generation 2, parents; G3, generation 3, children.

To assess whether our sample size provided adequate statistical power for the primary SEM analyses, we conducted Monte Carlo simulations in Mplus using parameter estimates from each of the four fitted models comparing the transdiagnostic anxiety factor with a disorder‐specific factor. Each simulation included 1000 replications with a sample size of 253, and we assessed (a) parameter and standard error bias, (b) 95% confidence interval (CI) coverage, and (c) statistical power, following the criteria proposed by Muthén and Muthén (2002). Across all four models, the primary path coefficients were statistically significant in nearly all replications (power range: 0.983–0.998), with 95% CI coverage ranging from 0.942 to 0.954. Parameter estimates and standard errors for this path showed minimal bias (all <6%), and all factor loadings demonstrated excellent power (≥0.999) and acceptable 95% CI coverage (0.939–0.961). These results met Muthén and Muthén (2002)'s three recommended simulation criteria: (a) parameter and standard error biases below 10%, (b) standard error bias for the target path below 5% (with a minor deviation in one model: −5.3%), and (c) 95% CI coverage within the 0.91–0.98 range.

### Comparative analysis of different parental transdiagnostic factors in predicting the offspring anxiety factor (secondary aim)

The goodness‐of‐fit statistics for the SEM analyses examining the predictive validity of different parental transdiagnostic factors on the offspring anxiety factor indicated acceptable to good model fit, with CFI and TLI values ranging from 0.828 to 0.920 and all RMSEA values below 0.05 (Table 2). The results indicated that all three parental transdiagnostic factors significantly predicted the offspring anxiety factor: parental internalizing (*β* = 0.469, SE = 0.111, *p* < 0.001), anxiety (*β* = 0.400, SE = 0.147, *p* = 0.006), and distress (*β* = 0.431, SE = 0.127, *p* = 0.001) (Table 3). Z‐tests revealed no statistically significant differences in the magnitude of standardized coefficients between internalizing and anxiety (*z* = 0.375, *p* = 0.708, 95% CI [–0.292, 0.430]), internalizing and distress (*z* = 0.225, *p* = 0.822, 95% CI [–0.293, 0.369]), and anxiety and distress (*z* = −0.160, *p* = 0.873, 95% CI [–0.412, 0.350]).

### Comparative analysis of different G2 transdiagnostic factors in predicting the onset of any childhood anxiety disorder in G3 (third aim)

Survival analysis results showed that all three G2 transdiagnostic factors significantly predicted the onset of any anxiety disorder before the age of 18 in G3: G2 internalizing (HR = 2.710, 95% CI: 2.702–2.718), anxiety (HR = 2.333, 95% CI: 1.950–2.715), and distress (HR = 2.111, 95% CI: 1.391–2.830) factors (Table 4). *z*‐tests indicated no significant differences in HR between the G2 transdiagnostic factors internalizing and anxiety (*z* = 1.774, *p* = 0.076, CI [–0.016, 0.315]); internalizing and distress (*z* = 1.379, *p* = 0.168, CI [–0.105, 0.605]); and anxiety and distress (*z* = 0.500, *p* = 0.617, CI [–0.292, 0.492]).

**TABLE 4:** Results for Cox regression models of the effect of change in parental transdiagnostic factors on the onset of offspring anxiety disorders.

G2 predictors	G3 Outcome	Estimates	S.E	*p*	HR	95% CI‐L	95%CI‐H
Internalizing factor		0.997	0.004	0.000	2.710	2.702	2.718
Anxiety factor	Any anxiety disorder onset in childhood	0.847	0.195	0.000	2.333	1.950	2.715
Distress factor		0.747	0.367	0.042	2.111	1.391	2.830

Abbreviations: CI, confidence interval; G2, generation 2, parents; G3, generation 3, children; HR, Hazard ratios.

## DISCUSSION

The primary aim of this study was to investigate the intergenerational transmission of anxiety using SEM and survival analysis. Our results provide three main findings: (1) anxiety was predominantly transmitted via a transdiagnostic pathway in which parents transmit *broad* vulnerabilities for different forms of anxiety disorders rather than *disorder‐specific* risk; (2) different parental transdiagnostic factors (i.e., internalizing, distress, and anxiety) significantly and similarly predicted the offspring's transdiagnostic anxiety factor; and (3) these transdiagnostic parental factors also significantly predicted the age of onset of childhood anxiety disorders. Taken together, these findings extend recent transdiagnostic work on familial transmission by testing shared versus disorder‐specific anxiety pathways within a latent‐variable framework and by translating these associations into time‐to‐onset risk estimates using survival analysis. Below, we discuss the implications of these findings.

Our findings suggest that anxiety is transmitted from parents to their children primarily through a transdiagnostic pathway, rather than through disorder‐specific mechanisms. Notably, power analyses indicated that the SEM models comparing these two pathways were sufficiently powered, suggesting that the non‐significance of the disorder‐specific pathway was not due to low statistical power. This aligns with prior research indicating that the intergenerational transmission of internalizing psychopathology is largely driven by broad, transdiagnostic vulnerabilities (Starr et al., 2014). Importantly, this pattern is also consistent with recent large‐scale cohort, registry, and meta‐analytic studies demonstrating that familial transmission of psychopathology is transdiagnostic (e.g., Chen et al., 2025; Uher et al., 2023; Zhou et al., 2023).

However, our results contrast with earlier studies that reported specificity in the familial aggregation of particular anxiety disorders. For instance, Fyer et al. (1993) found that relatives of individuals with social phobia were at significantly elevated risk for social phobia, but not for other anxiety disorders. Similarly, Telman et al. (2018) reported specificity in intergenerational transmission patterns, including strong mother–child concordance for social anxiety disorder and GAD. However, these studies were not designed to disentangle disorder‐specific variance from variance shared across anxiety disorders as was done herein. Thus, our findings offer a novel interpretation of intergenerational transmission patterns, suggesting that previously reported disorder‐specific associations may, in fact, reflect shared vulnerabilities common to anxiety disorders.

This pattern of transdiagnostic transmission is also consistent with findings from earlier genetic and family research on anxiety disorders. Andrews et al. (1990) found that while genetic factors contributed to broad neuroticism, there was no evidence for the inheritance of specific disorders. These findings suggest that genetic liability may confer a general predisposition to neurotic symptoms rather than disorder‐specific risk. Similarly, Schreier et al. (2008) found that maternal anxiety disorders predicted various types of anxiety disorders in offspring, indicating cross‐disorder transmission. In particular, maternal social phobia and GAD were associated with elevated risk for multiple anxiety outcomes in children, suggesting that these conditions may reflect a more general diathesis for anxiety that is transmitted across generations. Together, these findings appear to suggest that familial transmission of anxiety may reflect broad, transdiagnostic vulnerabilities rather than disorder‐specific risk. Consistent with recent work emphasizing broad psychopathology dimensions in intergenerational transmission (Zhou et al., 2023), our findings support the view that shared transdiagnostic vulnerability is a key feature of intergenerational anxiety risk.

Our findings also indicate that children's anxiety was predicted not only by parental anxiety but also by related transdiagnostic factors (i.e., internalizing and distress), each showing comparable predictive strength. This indicates that common underlying mechanisms across internalizing psychopathology may play a role in the intergenerational transmission of anxiety. These results align with the Hierarchical Taxonomy of Psychopathology framework (Kim & Eaton, 2015; Kotov et al., 2017; Krueger et al., 2018) and the tripartite model of anxiety and depression (Clark & Watson, 1991), both of which suggest that the overlap between anxiety and mood disorders can be attributable to a higher‐order internalizing factor or to negative affect. The finding that children's anxiety was also predicted by parental distress is consistent with evidence from Eaton et al. (2013), who demonstrated that both fear and distress dimensions predict subsequent fear factor by capturing shared vulnerabilities across internalizing conditions.

In addition, we found that parental internalizing, distress, and anxiety transdiagnostic factors significantly predicted the timing and likelihood of childhood anxiety disorder onset in offspring. Importantly, these findings extend the SEM results by quantifying the magnitude of risk, demonstrating how the hazard of developing an anxiety disorder increases with each standard deviation increase in parental transdiagnostic symptom levels. Consistent with prior findings (a) that parental internalizing symptoms significantly predicted children's anxiety (Fjermestad et al., 2020) and (b) that latent transdiagnostic internalizing factor played a central role in the development of internalizing disorders (Kessler et al., 2011), our findings underscore the importance of identifying parental transdiagnostic profiles associated with elevated risk during critical developmental periods.

By translating latent associations into interpretable hazard estimates, our survival analysis results provide clinically relevant information that can inform early identification and intervention for childhood anxiety. Specifically, (a) clinicians may benefit from assessing broad parental transdiagnostic symptoms, including anxiety, distress, and general internalizing symptoms, to identify children at elevated risk for developing anxiety disorders, and (b) interventions targeting broad underlying vulnerabilities may be especially effective in reducing the likelihood of anxiety disorder onset. Indeed, transdiagnostic prevention programs have shown promise in reducing anxiety symptoms: For example, the Unified Protocol for Transdiagnostic Treatment of Emotional Disorders in Children has demonstrated effectiveness in reducing not only anxiety but also broad internalizing symptoms, as shown in both a feasibility study (Caiado et al., 2022) and a randomized controlled trial (Ahadianfard et al., 2023; Caiado et al., 2024), with treatment gains maintained at follow‐up.

Our study has several strengths. One strength of our study is the use of a multigenerational sample that spanned several decades and included thorough assessments of various psychiatric disorders. This permitted a comprehensive analysis of the intergenerational transmission of anxiety disorders and the long‐term effects of parental psychopathology on offspring. By examining the comparative predictive validity of various transdiagnostic factors and comparing transdiagnostic versus disorder‐specific pathways, our findings provide novel insights into the nature of the intergenerational transmission of anxiety. In addition, the subsequent use of survival analysis after SEM to further examine whether transdiagnostic factors predict the onset of childhood anxiety disorders adds an important dimension to the analysis.

Our study has several limitations. First, our sample included offspring of probands with MDD. This may have contributed to higher rates of anxiety and mood disorders in both G2 and G3 than would be observed in population‐based samples. Although this may limit generalizability to community samples, higher base rates of internalizing conditions in this sample boosted indicator prevalence for SEM and event counts for survival models, which may have facilitated the detection of transdiagnostic effects. This may have also inflated the shared variance across transdiagnostic dimensions, given the strong correlation between anxiety and depressive disorders (Clark & Watson, 1991), potentially obscuring distinctions between anxiety‐specific and broader internalizing pathways. Second, because childhood anxiety onset was modeled using survival methods with right‐censoring, generalizability could be a concern if later‐adolescent estimates were based on a small or selected risk set. However, this concern is likely mitigated because most G3 participants (165/253; 65.22%) had complete childhood psychiatric histories, and the mean age at last interview in the full G3 sample was 14.97 years, indicating follow‐up typically extended through mid‐adolescence. Third, the predominately White sample limits the generalization of our findings to other racial and ethnic groups.

In summary, this study provides important insights into pathways by which anxiety is transmitted from parents to their children. The findings highlight the importance of transdiagnostic factors in the intergenerational transmission of anxiety and suggest that interventions and prevention should target these broad underlying factors. Future research should aim to replicate these findings in more diverse samples, to collect symptom‐level data to be able to capture the full spectrum of anxiety presentations (e.g., from subclinical to clinical), and to explore the underlying genetic, neurobiological, and environmental mechanisms that contribute to the intergenerational transmission of anxiety disorders.

## AUTHOR CONTRIBUTIONS

**Hyunsik Kim**: Conceptualization; methodology; formal analysis; writing—original draft; writing—review and editing. **Lifang Pan**: Methodology; formal analysis; supervision; writing—review and editing. **Ardesheer Talati**: Conceptualization; supervision; writing—review and editing. **Marc J. Gameroff**: Methodology; validation; writing—review and editing; project administration. **Myrna M. Weissman**: Writing—original draft; writing—review and editing; conceptualization; supervision; funding acquisition; resources. **Helen Blair Simpson**: Conceptualization; writing—original draft; writing—review and editing; supervision.

## CONFLICT OF INTEREST STATEMENT

In the last 5 years, M.R.W. has received research funds from National Institute of Mental Health, Brain and Behavior Foundation, The John Templeton Foundation, royalties from publications of books from Perseus Books, Oxford University Press, and the American Psychiatric Association Press, and royalties on the social adjustment scale from Multi‐Health Systems. None of these represent a conflict of interest. In the past 5 years, H.B.S. has received royalties from Cambridge University Press and UpToDate, Inc, served on a Scientific Advisory Board December 12, 2024 for Otsuka Pharmaceuticals, and receives a stipend from the American Medical Association for serving as Associate Editor for JAMA‐Psychiatry. None of these represent a conflict of interest. The remaining authors have declared that they have no competing or potential conflicts of interest.

## ETHICAL CONSIDERATIONS

All study procedures were approved by the Institutional Review Board of the New York State Psychiatric Institute/Columbia University (IRB approval date: February 12, 2025; approval number: 6145‐NYSPI). After providing participants with a complete description of the study, written informed consent was obtained from adults, and assent was obtained from minors along with written consent from their parents.

## Data Availability

Partial data are available at the NIMH Data Archive (NDA) for participants who gave consent during the 7th wave of data collection for their data to be deposited at the NDA. Data from the full sample at all study waves are only available when working with the P.I. because of the nature of participant consent when data were collected. Requests to access the full data are available from Dr. Adi Talati at adi.talati@nyspi.columbia.edu upon reasonable request.

## ENDNOTE

^1^
Missingness in childhood anxiety diagnostic data due to drop‐out was evaluated using Little's test for missing completely at random (MCAR), which indicated that the pattern of missingness was consistent with MCAR (*χ* ^2^ = 543.88, df = 495, *p* = 0.063).
